# Outcome of sutureless amniotic membrane dressing ring for acute Stevens–Johnson syndrome with severe ocular involvement: A case report of 5 patients

**DOI:** 10.1097/MD.0000000000047003

**Published:** 2026-01-09

**Authors:** Yuan-Kai Fu, Hsu-Ying Lin, Tsung-Ying Tsai, Wu-Yong Quan, Yi-Lin Liao, Yueh-Ju Tsai, David Hui-Kang Ma

**Affiliations:** a Department of Ophthalmology, Chang Gung Memorial Hospital, Taoyuan, Taiwan; b Sun-Ming Eye Clinic, Professional Eye Alliance, Taiwan; c Departments of Ophthalmology, Taipei Municipal Wanfang Hospital, Taipei Medical University, Taipei, Taiwan; d Department of Ophthalmology, Xiamen Chang Gung Hospital, Xiamen, China; e Department of Medicine, College of Medicine, Chang Gung University, Taoyuan, Taiwan; f Department of Chinese Medicine, College of Medicine, Chang Gung University, Taoyuan, Taiwan; g Center for Tissue Engineering, Chang Gung Memorial Hospital, Taoyuan, Taiwan.

**Keywords:** amniotic membrane dressing, ocular surface damage, Stevens–Johnson syndrome, toxic epidermal necrolysis

## Abstract

**Rationale:**

Stevens–Johnson Syndrome (SJS) and toxic epidermal necrolysis can lead to severe ocular damage and long-term vision complications. Our self-made sutureless amniotic membrane dressing (AMD) ring leverages the anti-inflammatory and regenerative properties of the amniotic membrane (AM), promoting healing, reducing scarring, and enhancing patient comfort.

**Patient Concerns:**

Five consecutive patients (3 females, 2 males; aged from 18 to 53 years) with acute SJS/TEN were at immediate risk of significant ocular surface damage, including corneal and conjunctival epithelial defects and eyelid margin erosions, which can progress to chronic dry eye, scarring, or permanent vision loss without timely intervention.

**Diagnoses:**

All patients were diagnosed with SJS/TEN based on their clinical presentations, including acute onset of mucocutaneous eruptions and extensive epidermal detachment, and comprehensive histories, confirmed by a dermatologist. Ocular involvement was assessed through detailed ophthalmologic examination and classified using the Sotozono grading system.

**Interventions:**

A self-made AMD ring, inspired by commercially available products such as Prokera, was used to cover the ocular surface. Constructed from readily available materials, this ring provides therapeutic benefits similar to Prokera without the need for suturing.

**Outcomes:**

High epithelial healing rates and stable follow-up visual acuity underscore this technique’s potential efficacy in selected SJS/TEN cases with severe ocular involvement.

**Lessons:**

Early and accessible interventions are essential for managing SJS/TEN-related ocular complications. The self-made AMD ring is a cost-effective and minimally invasive option that shows potential as an alternative to commercial AM devices, particularly in settings where availability or affordability is limited. Further research with larger cohorts is warranted to confirm its efficacy.

## 1. Introduction

Stevens–Johnson syndrome (SJS) and toxic epidermal necrolysis (TEN) are rare and severe mucocutaneous reactions characterized by extensive blistering and skin separation, often triggered by infections or adverse drug reactions.^[1]^ Despite their rarity, these conditions can lead to serious ocular and systemic complications, making treatment highly challenging.^[2]^

When SJS/TEN involves the ocular surface (cornea, conjunctiva, and eyelid margin), rapid keratinocyte apoptosis and inflammation can result in corneal epithelial defects, conjunctival sloughing, and pseudomembrane formation.^[3]^ Management focuses on controlling inflammation, preserving the ocular surface, preventing infection, and safeguarding limbal epithelial stem cells.^[4]^ However, due to the complexity of ocular surface disease associated with these conditions, new approaches are continually being explored to improve patient outcomes and reduce long-term complications.

Amniotic membrane dressing (AMD) has emerged as an effective therapeutic option for managing ocular surface disorders, including those related to SJS and TEN.^[5,6]^ The amniotic membrane (AM) derives from the innermost layer of the placenta, which is distinct from the chorionic layer surrounding the gestational sac. The AM contains a single epithelial layer, basement membrane, and avascular stromal matrix, conferring its anti-inflammatory and regenerative properties.^[7]^ These unique biological characteristics enable the AM to promote wound healing, reduce inflammation, and facilitate tissue regeneration.^[8]^

The use of AMD in SJS/TEN patients holds particular promise due to its ability to address multiple aspects of ocular surface damage, including epithelial defects, inflammation, and scarring.^[5,6]^ Furthermore, AMD provides a natural scaffold for tissue repair while minimizing the risk of immune rejection and infection.^[9]^

Traditionally, AMD requires suturing, which is technically challenging and can cause discomfort for patients.^[10]^ Self-retaining devices such as Prokera (Bio-Tissue, Inc., Doral, FL) provide sutureless AM application and have proven beneficial for acute ocular surface involvement.^[11]^ However, in many regions where the device is not available or delay in delivery is anticipated, to catch the treatment window period, alternative treatment is required.

In this article, we introduce a new technique using a self-made AMD ring and present the outcomes of ten eyes from 5 consecutive patients with SJS/TEN treated with this device.

### 1.1. Surgical method

Under sterile conditions, the periorbital skin was gently cleansed with povidone-iodine swabs, taking care to avoid excessive scrubbing that could damage the delicate epidermis, and sterile, nonadhesive towels were used to drape the area. The cryopreserved AM was thawed at room temperature and soaked for 3 minutes in a gentamicin solution (80 mg/10 mL BSS) and then spread onto a smooth surface. The stromal surface was placed face down, intended to serve as the contact side for the ocular surface, consistent with the orientation of the AM in the design of the Prokera.

**Figure 1.**
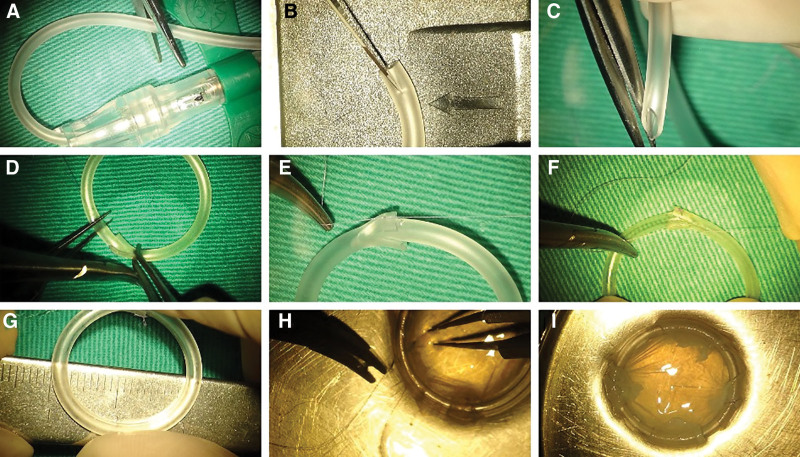
Construction of an AMD ring. (A) Using a 21-gauge infusion set, the tube was cut to the ideal length after the corneal diameter was measured. (B) At 1 end of the tube, a slit was made to cut the tube in the middle. (C) The opposite end was cut into a beveled plane. (D) A ring structure was formed by inserting the bevel into the blunt end. (E) The outer side of the tube was fixed with an 8–0 Vicryl suture. (F) In turn, the inner side of the tube was fixed again with an 8–0 Vicryl suture. (G) The completed frame provides a stable structure for AM to fixate. (H) The 4 corners of a square AM were folded over the edges of the ring and secured with 8–0 Vicryl mattress sutures. (I) A complete AMD ring with the epithelium side up is ready to use. AMD = amniotic membrane dressing.

After the diameter of the cornea was measured, an anti-symblepharon ring was created using the tube from a BD Vacutainer® UltraTouch™ Push Button Blood Collection Set (21G X 3/4 X 12 inches) (Fig. 1A). At 1 end of the tube, a slit was made across the tube diameter (Fig. 1B), and the other end was cut into a bevel plane (Fig. 1C) so that the bevel end could be inserted deep into the blunt end (Fig. 1D). The 2 ends were then fixed with 8–0 Vicryl sutures at the outer and inner sides of the tube (Fig. 1E and F), forming a ring with an outer diameter of approximately 21 to 23 mm (Fig. 1G) and approximately 10 mm wider than the cornea. The AM was spread underneath the symblepharon ring, and 4 equally spaced points were marked or visually determined. The 4 edges of the square AM were folded over the edges of the ring and secured at these points with 8–0 Vicryl mattress sutures (Fig. 1H). To insert the amniotic ring, the upper eyelid was gently retracted via a Desmarres retractor, and the ring was placed under the upper fornix, followed by placement into the lower fornix, ensuring full coverage of the entire damaged ocular surface. If a persistent epithelial defect of the cornea or conjunctiva was observed after the membrane dissolved, reapplication of the AMD ring was considered.

## 2. Case presentation

We retrospectively reviewed the electronic medical records of patients with SJS or TEN with ocular involvement who were treated at Chang Gung Memorial Hospital (CGMH), Taoyuan, Taiwan, between July 2019 and January 2024. The study adhered to the tenets of the Declaration of Helsinki and was approved by the Institutional Review Board of CGMH, Taiwan (Approval No. 202501447B0). Written consent was obtained from all patients or their legal guardians for the purpose of publication of case details and images. Ocular surface involvement was assessed using the Sotozono acute-stage ocular surface grading scoreocular surface grading score, with all recruited cases classified as grade 3, indicating corneal, conjunctival, or eyelid margin epithelial defects with pseudomembrane formation.^[3]^

Five patients with total of ten eyes were included, all treated with a self-made AMD ring. Clinical diagnoses, the culprit agents, preoperative ocular findings, time from symptom onset to AMD, and systemic treatments, including steroids or biologic drugs, were recorded. Demographics and preoperative findings are summarized in Table 1.

**Table 1:** Clinical presentation and medical treatment of the patients.

	Case 1	Case 2	Case 3	Case 4	Case 5
Age (y)	26	20	18	53	42
Sex	F	F	M	F	M
Diagnosis	SJS	TEN	TEN	SJS	SJS
Culprit	Zonisamide	Unknown (suspect viral infection)	Coxsackievirus A6	NSAID (Diclofenic)	Pembrolizumab
Preoperative findings (OU)	Corneal ED, Bulbar conjunctival ED, Lid margin erosion	Corneal ED, Bulbar and palpebral conjunctival ED, Pseudomembrane formation, Lid margin erosion	Corneal subtotal ED, Extensive bulbar and palpebral conjunctival ED, Pseudomembrane formation.	Bulbar and palpebral conjunctival ED, Diffuse SPK, Lid margin erosion	Corneal subtotal ED, Pseudomembrane formation, Lid margin erosion
Symptom to admission (days)	4	4	22	3	3
Hospital stays (months)	1.2	1.2	3.1	0.8	2.2
SCORTEN	0	3	2	2	3
Medical treatment					
Methyl-prednisolone	Yes	Yes	Yes	Yes	Yes
Etanercept	Yes	Yes	No	Yes	Yes
IVIG	No	No	Yes	No	No

ED = epithelial defects, SCORTEN = severity-of-Illness score for toxic epidermal necrolysis, SJS = Stevens–Johnson syndrome, SPK = superficial punctate keratitis, TEN = toxic epidermal necrolysis.

**Figure 2.**
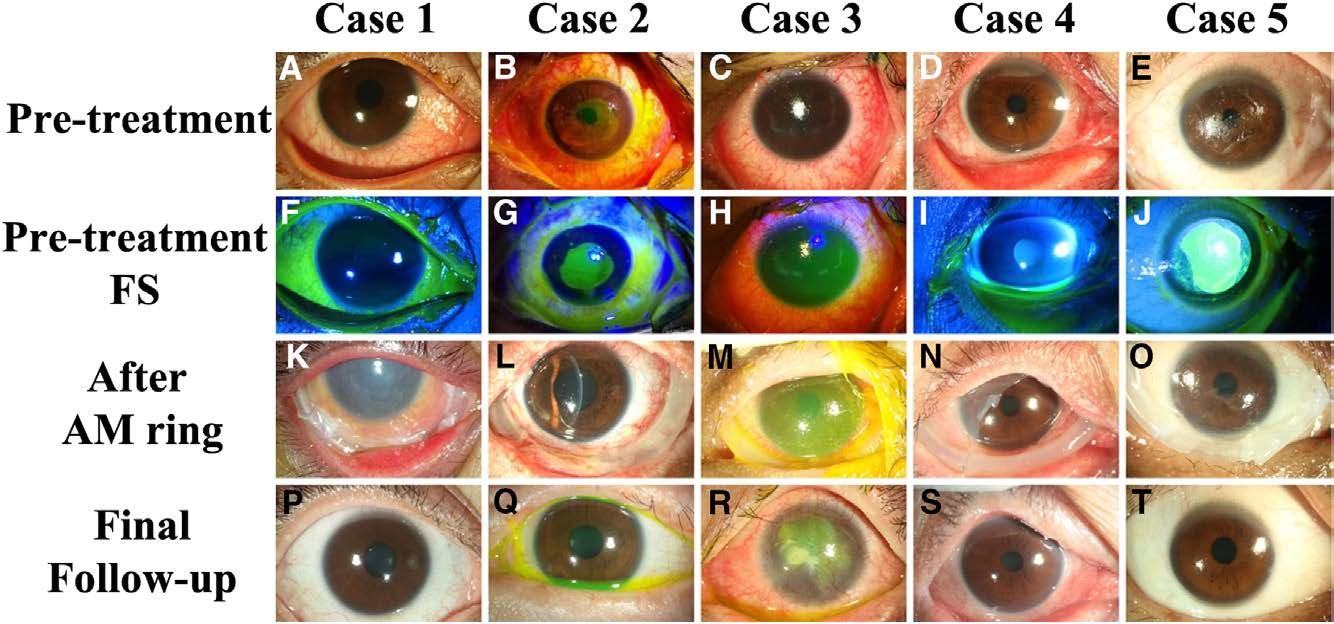
The clinical course of the same eye before and after AMD ring implantation for all 5 patients. The first row displays preoperative photos, followed by the second row for preoperative fluorescein staining images. The third row shows photos taken after AMD ring implantation, and the fourth row shows the clinical appearance at the final follow-up. Case 1 (A, F, K, P); Case 2 (B, G, L, Q); Case 3 (C, H, M, R); Case 4 (D, I, N, S); and Case 5 (E, J, O, T). AMD = amniotic membrane dressing, FS = fluorescein staining.

The outcomes of the 5 patients treated with the self-made AM ring are summarized in Table 2, and their clinical courses are illustrated in Figure 2. The cohort included 3 female and 2 male patients, with ages ranging from 18 to 53 years. All patients underwent at least 1 AMD, with repeated AMD procedures performed based on disease severity and inflammation grading. One patient received up to 5 AMD treatments.

**Table 2:** Clinical outcomes of the patients.

	Case 1	Case 2	Case 3	Case 4	Case 5
Days of AMD ring implantation after admission	4	8	22	10	6
Follow up (months)	7.1	46.2	10.6	43.6	1.2
Epithelialization in days after implantation (OD/OS)	31/35	24/29	NC/NC	13/13	37/NC
Additional operations	Repeated AMD (OU); OMET (OD)	Lacrimal duct reconstruction (OU)	AMD (OU) X 5 times; Allogeneic SLET (OU); OMET (OU)	OMET (OU); Punctal occlusion (OU)	AMD (OU) twice; Tarsorrhaphy (OS); Punctal occlusion (OU)
Initial BCVA (OD/OS)	20/25; 20/25	20/25; 20/25	20/50; 20/60	20/32; 20/60	20/40; 20/40
Final BCVA (OD/OS)	20/20; 20/25	20/20; 20/25	N/A	20/30; 20/30	N/A
Schirmer test (mm) (OD/OS)	2/3	12/7	28/31	3/18	N/A
Chronic Sotozono grading score (OD/OS)	2/2	2/1	21/19	5/5	2/6
Status	Alive	Alive	Expired	Alive	Expired

AMD = amniotic membrane dressing, N/A = not available, NC = not complete, OMET = oral mucosal epithelial transplantation, SLET = simple limbal epithelial transplantation.

Four patients were followed for more than 6 months posttreatment, while 1 patient expired approximately 1 month after admission. All patients received topical medications, including lubricants, anti-inflammatory agents (betamethasone 0.1% and tobramycin 0.3% + dexamethasone 0.1% ointment) and antibiotics (levofloxacin 0.5%), in addition to abundant lubrication. AMD ring was inserted once epithelial defect over cornea or bulbar conjunctiva was identified.

Regarding systemic treatment, all 5 patients received intravenous methyl-prednisolone (40 to 50 mg twice per day), and 4 received subcutaneous injections of etanercept, a TNF-α inhibitor. One patient also received intravenous immunoglobulin (IVIG). Despite all patients reaching OSGS grade 3, the extent of ocular surface involvement varied. Preoperative findings included: Case 3 had severe inflammation with extensive epithelial defect involving both cornea and conjunctiva but with minimal eyelid involvement; another 3 cases exhibited extensive conjunctival and corneal epithelial defects accompanied by eyelid margin erosion; and case 5 presented with severe corneal defects and eyelid erosion, while the conjunctiva remained relatively stable. During the follow-up period, 7 out of ten eyes with ocular surface defects healed, with a mean epithelial defect healing time of 26.0 ± 9.8 days. Three eyes from 2 patients remained unhealed at their last visit, and both patients expired due to worsening systemic conditions (cases 3 and 5).

In the chronic stage, 7 eyes had only minimal ocular involvement at the chronic stage, with scores ranging from 2 to 5 (2.71 ± 1.6), and 1 eye showed mild involvement with a final score of 6. In contrast, Case 3 presented with extensive, severe ocular involvement and poor response to multiple treatments. Despite receiving repeated AMD and additional procedures, including allogeneic simple limbal epithelial transplantation for corneal epithelial defect, epithelial healing was poor, and the patient demonstrated chronic scores of 21 and 29 for the right and left eye before he passed away. In general, final visual acuity in patients who could comply was preserved, with the last BCVA comparable to baseline levels. Dry eye syndrome was a significant concern. Among the 8 measured eyes, 2 experienced low tear production (Schirmer test results <5 mm).

### 2.1. Representative cases

#### 2.1.1. Case 1

A 26-year-old Han Chinese woman with a history of post-traumatic seizures treated since age 12 developed a fever and skin rash characterized by scattered maculopapular eruptions, atypical flat targets, bullae formation, and oral and genital erosions, covering 5% to 10% of her total body surface area. She also experienced bilateral ocular irritation and sought care in our emergency department 4 days after symptom onset. Following consultation with a dermatologist, she was diagnosed with SJS, with Zonisamide, an antiepileptic drug, identified as the culprit. Her initial ophthalmologic exam revealed eyelid margin erosion and conjunctival defects with pseudomembrane formation, though her corneas were relatively clear, without epithelial defects (Fig. 2A and F). She was initially managed conservatively with topical eye drops and lubricants.

However, on the fourth day of admission, corneal involvement was detected, necessitating the application of an AMD ring (Fig. 2K). She was regularly followed up, and a repeated AMD ring procedure was performed 10 days after the initial application. Significant improvement in her ocular condition was observed, with healing noted in the right eye by day 31 and in the left eye by day 35 postimplantation. Approximately 1 month later, she underwent conjunctival reconstruction with oral mucosa for right eye. Her visual acuity was preserved, and after 7 months of follow-up, her eyes were normal (Fig. 2P), and the chronic Sotozono score was only 2 in both eyes.

#### 2.1.2. Case 2

A 20-year-old Han Chinese female with no underlying medical conditions developed a persistent fever and skin rash, accompanied by oral erosions, after taking an unknown medication for a common cold. Upon arrival at the emergency department, she was diagnosed with TEN, with a total body surface area involvement of approximately 36% and was treated in the burn ICU. She also experienced bilateral eye discomfort, presenting with diffuse superficial punctate erosions in both eyes and thick pseudomembranes on both the upper and lower palpebral conjunctiva. Her symptoms worsened, leading to significant corneal and conjunctival epithelial defects (Fig. 2B and G). An AM ring was applied 1 week after symptom onset to protect the ocular surface.

During the follow-up period, her general condition improved, and her corneas healed well, with the right eye healing by day 24 and the left eye by day 29 postoperation (Fig. 2L). However, she developed lacrimal duct stenosis, necessitating lacrimal duct reconstruction surgery for both eyes. Her condition remained stable, and she continued to be monitored. At her last visit, nearly 3 years later, her chronic Sotozono score was 2 for the right eye and 1 for the left eye (Fig. 2Q).

#### 2.1.3. Case 3

An 18-year-old Han Chinese teenage boy developed a fever and a generalized skin rash, which began on the extremities and spread centrally, along with oral involvement. He was transferred to our hospital 22 days after the onset of symptoms. Before transfer, he had been treated with intravenous immunoglobulin, but no eye treatment was provided at the previous hospital. A review of his medical history revealed no specific culprit drug, but a throat swab PCR test showed positive results for Coxsackie virus A6, which the dermatologist identified as the most likely cause. He was admitted to the burn intensive care unit for specialized care.

Upon arrival at the emergency department, an ophthalmologist was consulted due to bilateral ocular irritation and multiple periorbital skin lesions that had persisted for days. Although no eyelid erosion or symblepharon was observed, extensive epithelial defects involving both cornea and conjunctiva were noted (Fig. 2C and H). In response, an emergent AMD using a self-made AM ring was applied to protect his ocular surface, with daily ophthalmologic evaluations. Despite this intervention, there was limited response in reepithelialization, and the AMD ring was applied an additional 4 times over the course of 1 month following the initial treatment (Fig. 2M).

The patient’s general condition improved, and he was transferred back to the dermatology ward, eventually being discharged about 3 months after admission. However, during outpatient follow-up, poor corneal epithelial healing with corneal conjunctivalization necessitated bilateral cadaveric allogeneic simple limbal epithelial transplantation and subsequent ocular surface reconstruction with oral mucosa and AMD with full ocular surface coverage. Unfortunately, the patient’s general condition deteriorated due to TEN-related bronchiolitis obliterans and respiratory failure. Because the patient and family declined lung transplantation, he suffered from pneumonia, which lead to sepsis and acute respiratory distress, and he eventually passed away.

## 3. Discussion

Both SJS and TEN are immune-mediated disorders affecting the skin and mucous membranes, often involving the ocular region and resulting in severe, long-lasting visual complications. In the acute phase, these conditions are characterized by rapid-onset keratinocyte apoptosis, followed by inflammation and extensive loss of ocular surface epithelium, including the corneal, conjunctival epithelium, and eyelid margin skin.^[3]^ Ocular involvement can range from mild conjunctival hyperemia to near-total sloughing of the entire ocular surface epithelium. Managing these complications is extremely challenging, with the primary focus being to halt inflammation, preserve ocular surface integrity, prevent infection, and protect limbal epithelial stem cells.^[12]^ Early application of AM therapy has been shown to prevent the devastating consequences.^[13]^

AMD show significant potential as a therapy for ocular surface diseases associated with SJS/TEN, offering improvements in both vision and life quality. To minimize delay in treatment and facilitate timely intervention, products like Prokera and Prokera Slim (Bio-Tissue, Inc., Doral, FL) have been introduced.^[11]^ Prokera reduces the need for sutures onto the ocular surface and easing the discomfort associated with conventional AMD, while offering effective treatment. However, Prokera’s high cost and limited availability in underdeveloped regions are obstacles to widely use this device.^[14]^

To address these challenges, a customized self-made AMD ring constructed with an infusion tube offers a more accessible and cost-effective alternative. The cost of 1 AM ring at our hospital includes the processing and preservation of the harvested AM in a tissue bank, as well as materials such as the IV set and 8-0 Vicryl used for the frame, totaling around USD 100, which is ten times less than the price of Prokera imported to Taiwan (more than USD 1000 at present currency rate). The surgical technique is simple, making it easy to use not only in operation room but also at bed sides. Previous studies have described the use of customized symblepharon rings made from IV tubing or nasogastric tubes; however, these approaches do not involve attaching the AM directly onto the ring, and they still require fixation of the AM to the eyelid margin or ocular surface.^[15,16]^ Another related method involves ring-supported AM constructs for chemical burn injuries, in which the AM is sutured only along the outer rim of the ring, leaving the central area uncovered.^[17]^ In this case series, we present the long-term outcomes of 10 eyes from 5 consecutive patients with SJS or TEN who were treated with our fully assembled, self-retained AMD ring technique, providing insight into its potential as a feasible and practical therapeutic option for this patient population.

In this study, 7 out of ten eyes in patients with SJS/TEN achieved full epithelial healing, showing a promising success rate higher than the 59% reported by Zhou et al for ocular surface disease patients treated with Prokera.^[14]^ Due to the variability of the patients’ general conditions and disease severity, 2 patients expired from complications related to SJS/TEN, and both had poor ocular wound healing. The remaining 3 patients had long-term follow-ups with unchanged visual acuity similar to their baseline levels. However, dry eye syndrome remained a major long-term issue, with 3 patients showed poor Schirmer test values, requiring lacrimal duct orifice occlusion. A major disadvantage of this technique is that since AMD ring can only protect the cornea and bulbar conjunctiva, in 3 patients conjunctival keratinization developed and required oral mucosal reconstruction. For patients with palpebral conjunctival involvement, and notably lid margin skin erosion, total globe AM coverage and fixation with Histoacryl glue described by Shanbhag et al should be adopted.^[6]^

This case series is limited by its small sample size due to the rarity of SJS/TEN, and outcomes may vary with disease severity and comorbidities. Previously, we reported that the severity of acute-stage manifestations correlates with the severity of chronic sequelae, and that the average chronic severity scores in patients receiving AM transplantation within 7 days of onset were significantly lower than in those who received it after 7 days.^[18]^ Although short-term surrogate outcomes (epithelial healing, BCVA) may not predict long-term ocular integrity or patient quality of life, timely AMD may offer patients in need the best opportunity for long-term recovery. Additionally, as this was a retrospective case series of a rare and acute condition, the potential for selection bias cannot be excluded. Larger, prospective studies are needed to confirm efficacy and refine the technique for broader ocular surface involvement.

## 4. Conclusion

We introduced a self-made AMD ring using an infusion tube is a cost-effective, minimally traumatic option for treating severe ocular involvement in SJS/TEN. It may also benefit patients with chemical burns restricted to the cornea or bulbar conjunctiva. The simplicity and regenerative properties of AM make this approach a valuable alternative in settings where commercial products are inaccessible or cost-prohibitive.

## Author contributions

**Conceptualization:** Yueh-Ju Tsai, David Hui-Kang Ma.

**Data curation:** Yuan-Kai Fu, Tsung-Ying Tsai, David Hui-Kang Ma.

**Funding acquisition:** David Hui-Kang Ma.

**Investigation:** Yuan-Kai Fu, Tsung-Ying Tsai.

**Methodology:** Wu-Yong Quan.

**Project administration:** David Hui-Kang Ma.

**Resources:** Tsung-Ying Tsai, Yi-Lin Liao, David Hui-Kang Ma.

**Software:** Yueh-Ju Tsai.

**Supervision:** Yi-Lin Liao, Yueh-Ju Tsai, David Hui-Kang Ma.

**Validation:** Hsu-Ying Lin, Yi-Lin Liao, David Hui-Kang Ma.

**Visualization:** Yuan-Kai Fu.

**Writing – original draft:** Yuan-Kai Fu, Hsu-Ying Lin.

**Writing – review & editing:** David Hui-Kang Ma.

## References

[1] Ueta M. Findings by an International Collaboration on SJS/TEN with severe ocular complications. Front Med (Lausanne). 2021;8:649661. 34926478 10.3389/fmed.2021.649661 PMC8672139

[2] Chen YL Tsai TY Pan LY. Ocular manifestations and outcomes in children with Stevens-Johnson syndrome and toxic epidermal necrolysis: a comparison with adult patients. Am J Ophthalmol. 2023;256:108–17. 37633318 10.1016/j.ajo.2023.08.009

[3] Sotozono C Ueta M Nakatani E.; Japanese Research Committee on Severe Cutaneous Adverse Reaction. Predictive factors associated with acute ocular involvement in Stevens-Johnson syndrome and toxic epidermal necrolysis. Am J Ophthalmol. 2015;160:228–37.e2. 25979679 10.1016/j.ajo.2015.05.002

[4] Pan LY Wang CW Tsai TY. Post hoc analysis of role of etanercept in ocular sequelae of Stevens-Johnson syndrome/toxic epidermal necrolysis. Ophthalmology. 2024;131:864–6. 38556174 10.1016/j.ophtha.2024.03.023

[5] Gregory DG. Treatment of acute Stevens-Johnson syndrome and toxic epidermal necrolysis using amniotic membrane: a review of 10 consecutive cases. Ophthalmology. 2011;118:908–14. 21440941 10.1016/j.ophtha.2011.01.046

[6] Shanbhag SS Hall L Chodosh J Saeed HN. Long-term outcomes of amniotic membrane treatment in acute Stevens-Johnson syndrome/toxic epidermal necrolysis. Ocul Surf. 2020;18:517–22. 32200005 10.1016/j.jtos.2020.03.004 PMC7811362

[7] Tseng SC Espana EM Kawakita T. How does amniotic membrane work? Ocul Surf. 2004;2:177–87. 17216089 10.1016/s1542-0124(12)70059-9

[8] Kogan S Sood A Granick MS. Amniotic membrane adjuncts and clinical applications in wound healing: a review of the literature. Wounds. 2018;30:168–73. 30059334

[9] Malhotra C Jain AK. Human amniotic membrane transplantation: different modalities of its use in ophthalmology. World J Transplant. 2014;4:111–21. 25032100 10.5500/wjt.v4.i2.111 PMC4094946

[10] Kotomin I Valtink M Hofmann K. Sutureless fixation of amniotic membrane for therapy of ocular surface disorders. PLoS One. 2015;10:e0125035. 25955359 10.1371/journal.pone.0125035 PMC4425509

[11] Kolomeyer AM Do BK Tu Y Chu DS. Placement of ProKera in the management of ocular manifestations of acute Stevens-Johnson syndrome in an outpatient. Eye Contact Lens. 2013;39:e7–11. 22683916 10.1097/ICL.0b013e318255124f

[12] Kohanim S Palioura S Saeed HN. Acute and chronic ophthalmic involvement in Stevens-Johnson syndrome/toxic epidermal necrolysis – a comprehensive review and guide to therapy. II. Ophthalmic disease. Ocul Surf. 2016;14:168–88. 26882981 10.1016/j.jtos.2016.02.001

[13] Mortensen XM Shenkute NT Zhang AY Banna H. Clinical outcome of amniotic membrane transplant in ocular Stevens-Johnson syndrome/toxic epidermal necrolysis at a major burn unit. Am J Ophthalmol. 2023;256:80–9. 37598739 10.1016/j.ajo.2023.07.026

[14] Zhou TE Robert MC. Comparing ProKera with amniotic membrane transplantation: indications, outcomes, and costs. Cornea. 2022;41:840–4. 34483269 10.1097/ICO.0000000000002852

[15] Lam SS Sklar BA Schoen M Rapuano CJ. Severe ocular alkali injury managed with an externally sutured amniotic membrane and customized symblepharon ring. Taiwan J Ophthalmol. 2023;13:101–5. 37252174 10.4103/2211-5056.362597 PMC10220433

[16] Ceylan A Mergen B Aydin FO Avci E Yildirim Y. Sutureless amniotic membrane transplantation using pediatric nasogastric tube for patients with acute Stevens-Johnson syndrome/toxic epidermal necrolysis. Eye Contact Lens. 2023;49:199–203. 36943174 10.1097/ICL.0000000000000986

[17] Dogan C Arslan OS Ozdamar A Mergen B Sarici AM Iskeleli G. Efficacy of fixation of the amniotic membrane on a symblepharon ring with continuous suturing in acute ocular chemical burn patients. Int Ophthalmol. 2019;39:2103–9. 30467665 10.1007/s10792-018-1049-1

[18] Ma DH Tsai TY Pan LY. Clinical aspects of Stevens-Johnson syndrome/toxic epidermal necrolysis with severe ocular complications in Taiwan. Front Med (Lausanne). 2021;8:661891. 34055837 10.3389/fmed.2021.661891 PMC8149748

